# Enhancing the Adult and Paediatric Palliative Care System: Spanish Professionals’ and Family Caregivers’ Suggestions for Comprehensive Improvement

**DOI:** 10.3390/healthcare12010065

**Published:** 2023-12-27

**Authors:** Laura Llop-Medina, Paula García-Muñoz, Francisco Ródenas-Rigla, Jorge Garcés-Ferrer

**Affiliations:** Polibienestar Research Institute, University of Valencia, 46022 Valencia, Spain; garmuoz7@uv.es (P.G.-M.); francisco.rodenas@uv.es (F.R.-R.); jordi.garces@uv.es (J.G.-F.)

**Keywords:** palliative care system, adults palliative care, paediatric palliative care, quality improvement, evaluator indicators, family caregivers, healthcare professionals

## Abstract

This research critically explores deficiencies in the palliative care system, focusing on evaluation and treatment aspects for both adult and paediatric patients. Using a qualitative methodology, the study engages healthcare professionals and family caregivers to uncover perspectives on the existing state of palliative care. Conducted through three focus groups and a semi-structured in-depth interview with participants recruited from Virgen de la Arrixaca University Clinical Hospital, this research illustrates critical issues, highlighting the insufficient healthcare workforce and resources to meet the comprehensive needs of patients and their families. Recommendations include holistic care addressing social, emotional, psychological, socio-familiar, and economic dimensions, supported by embedded support groups and the enforcement of relationships with palliative associations. This study also advocates for improved health institutional coordination, social worker support, and ongoing health professional satisfaction monitoring. In paediatric care, specific demands involve specialised units, medical team continuity, 24 h paediatrician care, and a more professional paediatric approach. Beyond problem identification, this study offers valuable insights for shaping health policies and tools, incorporating new indicators and introducing grief bereavement support in clinical reports, contributing to the advancement of patient evaluation in palliative care.

## 1. Introduction

Palliative care, as defined by the World Health Organisation [1], aims to enhance the quality of life and alleviate suffering for patients with life-threatening illnesses and their families. This approach involves early detection, appropriate symptom management [2,3] and comprehensive care that addresses physical, emotional, social, spiritual, and psychological needs through the collaboration of an interdisciplinary team [4,5].

However, palliative care faces challenges in practice. Both families and patients are constrained by the capacities of the health care system, resulting in long waits due to long standby lists, compounded by a shortage of professionals and insufficient resources in palliative care [6,7]. This situation generates a greater burden for family caregivers, who lack the training and information necessary to provide this care [6], and professional care is limited to the terminal phase of the disease [3,4,5,8]. In short, this reality harms the physical, social, and psychological well-being of both patients and family caregivers.

Limited access to palliative care, stemming from resource scarcity and its subsequent effects on the timeliness and quality of assistance, results in underutilised palliative care units [9,10,11]. The consequence of this underutilisation is an augmented fatality rate. Studies [11,12] have highlighted the significantly shortened duration from the initial palliative consultation to the time of death for adult palliative patients due to this issue. These studies remark on the effect on surgical palliative patients; other researchers have found similar results in other adult palliative patients [13]. In the case of paediatric palliative patients, a study centred on Spain disclosed an alarming rate of mortality of children and adolescents diagnosed with life-threatening illnesses [9]. This underscores the critical impact of restricted access to palliative care services, particularly in the paediatric demographic, emphasising the urgency for comprehensive improvements in palliative care accessibility and delivery.

Considering this challenging scenario, it becomes imperative to take decisive actions to address the needs of both adult and paediatric patients requiring palliative care. This necessitates attention from the moment of diagnosis and patient assessment through the treatment of their needs, extending beyond the purely physical aspects.

In this paper, our focus is directed towards the region of Murcia, Spain, which boasts a population of 462,979 inhabitants [14], making it the seventh most populated municipality in Spain. With shared competencies in health matters with the government, this region is served by the Virgen de la Arrixaca University Clinical Hospital in Murcia (VAUCH), the largest hospital complex in the Murcia public health system. VAUCH has a comprehensive history of delivering palliative care, bereavement support, and end-of-life care, and it has actively engaged in researching the effectiveness of these services [15]. To address these challenges, the main objective of this research is to analyse the elements that influence the quality of palliative care provision from the perspective of professionals and family caregivers, considering the use of certain scales by professionals and the perceptions of deficiencies and proposals for improvement by both stakeholders. This analysis aims not only to point out the deficiencies but also to provide enhancement suggestions for a more effective and comprehensive palliative care system.

## 2. Materials and Methods

This research employs a qualitative methodological design with the overarching aim of comprehensively studying the perspectives of family members of patients in palliative care and healthcare professionals regarding the palliative care system. The focus is on identifying their perceptions of existing deficiencies and areas for improvement, ultimately proposing recommendations in the treatment and evaluation areas.

Three focus groups were designed for this purpose. The first two involved healthcare professionals, and the third was conducted with family carers of palliative care patients. In addition, a semi-structured in-depth interview component was integrated into the fieldwork to further explore the nuanced views and active participation of families.

All the fieldwork for this study was carried out at VAUCH. The dedicated healthcare team at VAUCH played a crucial role in assisting with the research, and this paper presents findings that emerged from the collaborative efforts with their team, leveraging their extensive experience and expertise in these specialised areas.

This qualitative paradigm facilitated a profound exploration of perspectives on the quality improvement of the palliative system in general, as well as understanding how they measure the quality of palliative patients and how we can improve the way they assess it.

Ethical approval for the development of this research has been obtained from the Ethics Committee on Research of VAUCH (ref. 2020-9-3).

### 2.1. Identification of Participants

Three focus groups and a semi-structured in-depth interview were designed to study the perspectives of quality care on palliative care services. Two of the three focus groups were designed to attend the professional standpoint: doctors, hospital managers, nurses, and paediatricians from the region of Murcia. Each focus group was composed of 4 persons. The third focus group, with three participants, was focused on the viewpoint of families caring for palliative care patients. 

The selection of techniques in this research employed a purposive sampling approach, a deliberate choice driven by the sensitive nature of the study and the inherent challenges in accessing the stakeholders involved—busy healthcare practitioners and family caregivers of palliative patients. The intricacies of this research field not only justify the specific number of participants but also account for the deliberate lack of variability in our sample, as outlined in Table 1. The sampling method as the size of our sample is also justified by previous studies in the health field [16,17], as well as by the principle that the transferability and dependability of the data are influenced by the comprehensive description of all possible contextual factors impacting the inquiry [18]. A sample that is fully contextualised helps prevent unwarranted generalisation. To accomplish the best description of the context of this research, we have followed the COREQ guideline [19] and we apport 32 criteria of our research in the Supplementary Materials (Table S1).

The inclusion of a semi-structured in-depth interview served a dual purpose: to augment the richness of qualitative data and, more critically, to adhere to a standard of scientific rigour by enhancing the symbolic representation within the research framework. This approach ensures a nuanced and comprehensive exploration of the perspectives of the participants, contributing to the depth and validity of the study’s findings.

The enrollment of the participants in the research was facilitated by health workers of VAUCH between October and November of 2021. 

To identify the participants in the verbatims, the following coding has been established: HP (health professionals) and FC (family caregivers). FC1 to FC3 corresponds to focus group participants, and FC4 corresponds to the case study of the only family member with a patient in a paediatric palliative care unit. Within the group of professionals, we distinguish HM (Health managers), M (medicine profile) and N (nursing profile).

### 2.2. Data Collection

The research design encompassed three focus groups conducted at Murcia’s University due to its well-equipped facilities and proximity to VAUCH. Each focus group had a duration ranging between 60 and 90 min. Meanwhile, the semi-structured in-depth interview had a duration of around an hour.

Before the commencement of the focus groups and the in-depth interview, a comprehensive set of open-ended questions, categorised by relevant topics, was meticulously designed for each technique. Both family caregivers and healthcare professionals were invited to share their perspectives about the deficiencies of the palliative care system, as well as to provide insights and recommendations for its improvement. In addition, healthcare professionals were specifically asked about academic and professional evaluation indicators in palliative care, delving into aspects such as their application within the hospital setting and eliciting their opinions on the sensitivity and appropriateness of these indicators in capturing the diverse dimensions of palliative care. This meticulous approach ensured a thorough exploration of the perspectives of both groups, enriching the qualitative data and contributing to a comprehensive understanding of the palliative care landscape.

All focus groups were conducted with meticulous attention to ethical standards, obtaining informed consent from participants before proceeding. The sessions were digitally recorded to ensure accurate capture of discussions and subsequently transcribed. The intent behind this comprehensive documentation was to facilitate in-depth data analysis, a task entrusted to an experienced researcher well-versed in the subject matter and adept in qualitative research methodology. This methodological rigour was employed to derive meaningful insights from the rich tapestry of perspectives and experiences shared during the focus groups, contributing to the robustness of the research findings.

### 2.3. Data Analysis

Following the implementation of research techniques, a rigorous analysis of the qualitative data ensued. Two members of the research team, well-versed in the subject matter and guided by perspectives of value-based care [19] and patient and family-centred [20] approaches, undertook the codification of the qualitative data, and undertook the codification of the data. In a posterior phase, the researchers discussed the codes, arriving at an interpretative agreement of focus groups and the interview transcripts by exploring the connections and associations between codes and categories. The result was the emergence of overarching themes and sub-themes, as illustrated in Table 2. This thematic analysis was driven by the interpretative objective of the study and the inherent limitations within the sample.

To enhance the efficiency of this intricate coding process, we leveraged the capabilities of Atlas.ti software version 9. 

### 2.4. Trustworthiness

The methodological rigour of this paper is underscored by a comprehensive research methodology incorporating ethical considerations. Before the start of the fieldwork, this research was designed with the support and collaboration of the VAUCH team and with the approval of its ethics committee. The committee played a central role in guiding and approving the ethical premises, emphasising the protection of participants’ rights and confidentiality, and considering the sensible object of study.

The collaboration with professionals of VAUCH was fundamental during the recruitment phase to access a very sensible sample of familiars and busy professionals.

To allow a structured and focused exploration of the research objectives, the research team elaborated a discussion group and interview script. Two proficient members of the research team conducted the focus groups and participated in the codification and discussion process to ensure a robust and reliable analysis. The inclusion of the Consolidated Criteria for Reporting Qualitative Studies (COREQ) 32 checklist was used to demonstrate a commitment to transparency as well as ensure the most systematic approach possible to data analysis, considering the evident bias given the insufficient heterogeneity and size of the sample and the own characteristics of VAUCH [21] (Supplementary Table S1).

## 3. Results

In this research, we explored recommendations for enhancing the palliative care system by focusing on the evaluation of the assistance of palliative patients (adults and paediatrics). Through initial three focus groups and an in-depth interview with healthcare professionals and family caregivers, we gained insights into proposals for improving the quality of the care palliative system.

### 3.1. Palliative Care Scales

To delve into the recommendations for improving the palliative care system, it is essential to focus on how healthcare professionals evaluate their palliative patients. The focus groups with healthcare professionals shed light on various evaluation indicators, offering contextual information on their usage, the appropriateness of their dimensions and the adequacy of their application in assessing palliative care patients. Among the main scales utilised and known by the clinical team at VAUCH, they were asked about some scales: Karnofsky, ECOG, Edmonton, Gijón, and FAMCARE. Notably, healthcare professionals at VAUCH emphasised that while they employ some of the mentioned scales, the Karnofsky and ECOG are the primary choices. Among these, ECOG is favoured from a feasibility point-of-view: this scale is brief, so it is easier to apply due to the unit time-attention constraints. As one professional explained, “*We tend to write ECOG rather than Karnofsky because the assessment is much longer, so we use ECOG, which is shorter*” (HP, M5).

However, it is acknowledged that despite the usage of these scales for palliative patient evaluation, they may not be the most suitable for this patient population. The ranges of the scales are viewed as somewhat general and may either overlook the specific challenges faced by the palliative patient or focus solely on a particular palliative group, the ones that are in an outpatient phase.

“*ECOG is not very suitable because it is too broad a scale, it is much better to use the Karnofsky or the PTS because the sections of ECOG correspond to two or three sections of the Karnofsky, so it is too broad and not very specific for palliative issues… so I think ECOG should not be used in palliative issues because it is too general…*”

(HM, M7).

“*…perhaps Karnofsky is more for palliative patients who are still in an ambulatory phase…the Karnofsky scale is used in the usual oncological patient…[…]*”

(HM, M8).

This insensitivity to attending to the particularities that palliative patients are facing is not exclusive to Karnofsky or ECOG; it affects widely used scales such as Edmonton and Gijón ones. The lack of sensitivity to address the specific challenges faced by palliative patients is not confined to scales like Karnofsky or ECOG; it extends to other widely utilised measures such as Edmonton and Gijón. This insensitivity is attributed to the omission of palliative symptoms such as insomnia, delirium, or asthenia from the evaluation, as well as the certain dimensions (e.g., socio-familiar or economic aspects) in assessing the sick; or the trickiness of estimating some of their dimensions.

“*I think that in general, the Edmonton scale is complete, but… there may be situations where it doesn’t fit or there may be some symptoms that are not included…*”

(HM, M7).

“*[about Edmonton scale] There are some symptoms that are also frequent and that are not there… here he does not talk about delirium or insomnia… I do miss the nocturnal delirium quite a lot. I also miss the symptom of asthenia which is also very prevalent and very disabling*”

(HM, M8).

“*Yes, at home we have the Gijón scale in the computerized history, but I don’t like it, I don’t think it identifies socio-familial or economic needs… it is a bit cumbersome in some items… I don’t think it is the most appropriate, no, nor do I know which other scale could replace it… the social, socio-familial, and economic dimension is very broad and very important, perhaps here the social workers would provide us with much more*”

(HM, M7).

“*As for the socio-family scale of Gijón, it is not specific to palliative care, and I think it’s good that you don’t reflect it*”

(HM, M8).

Finally, healthcare professionals express mixed views on the appropriateness of using the FAMCARE scale in assessing satisfaction with palliative care. While some suggest its utilisation as a valuable tool for evaluating care satisfaction and initiating improvement cycles, others argue against its current application. One notable critique is the absence of an item assessing whether patients and their families have received empathetic assistance from healthcare professionals. This oversight, according to dissenting voices, represents a crucial dimension in palliative care that directly influences overall satisfaction. The recommendation to add an item related to the provision of empathetic care underscores the importance of capturing the emotional and relational aspects of the patient-caregiver-professional dynamic. This nuanced perspective from healthcare professionals highlights the ongoing dialogue and refinement needed in the selection and application of evaluation tools like the FAMCARE scale in the complex landscape of palliative care.

Above the mixed opinion, healthcare professionals lean towards the FAMCARE scale, which is potentially the most effective evaluation indicator for palliative care patients, particularly with suggested modifications. This viewpoint emphasises the importance of tailoring evaluation tools to the unique needs and nuances of palliative care, acknowledging that a one-size-fits-all approach may not be suitable. The proposed modifications likely aim to enhance the scale’s sensitivity to the specific challenges and dimensions relevant to palliative care, emphasising the holistic nature of patient and family needs. This professional perspective signals a recognition of the FAMCARE scale’s potential to capture a more comprehensive understanding of the palliative care experience, making it a valuable candidate for refining the assessment process in this critical healthcare domain (Table 3).

### 3.2. Advancing Palliative Care for Adults: Some Improvement Suggestions

In the context of advancing care for adults, several improvement suggestions emerged from the insights gathered from the focus groups that included professionals and families affected by palliative care. These insights contribute to the policy debates regarding healthcare system enhancements, specifically for adult palliative and their families' lives. The recommendations presented below correspond to the results obtained from the three discussion groups.

#### 3.2.1. Lack of Resources and the Need to Increase the Headcount of Workers and Dimensions in Palliative Care Units

Professionals voiced some common demands for improving the working conditions of healthcare providers. They emphasised the constrained time and available resources they dispose of to attend to their patients, often resulting in the prioritising of certain tasks over others. More particularly, their time tends to be focused on addressing the physical symptoms reported by patients, which can lead to the neglect of patients’ psychological needs and comprehensive evaluation.

“*…I think that tackling everything…it gives me a shock…but if you put a scale and we start little by little and the system helps us with another team of doctors and nurses…but right now we work…in a state of hierarchical prioritization…*”

(HP, N1).

“*I would prioritize the physical, what the symptoms are and that the symptoms are controlled and then the social, I mean, if a patient is in pain, has physical suffering, I think that would be the first thing to prioritize*”

(HP, N6).

The specific demand or suggestion arising from this discussion is crystal clear: an increase in the workforce would directly impact patient care overall given the complex and demanding need of palliative care patients. Professionals call for not only more healthcare staff but also the addition of social workers who could help in the evaluation tasks. Families also demand the help of social workers and highlight the lack of economic and socio-familiar support within palliative care.

“*…There has to be someone else on top to follow up… I don’t care who it is, but there is also a lack of follow-up, I have no complaints about palliative care, but financially, well, one must deal with the family*”

(HM, M7).

“*…Not only if he has support, but also where he lives, with whom he lives…how this patient is social, is the dimension that needs to be assessed with the social workers…[…]*”

(HP, M4).

The psychological, socio-familiar, and economic dimension is totally forgotten because of the lack of resources and workforces. Addressing these resource shortages by increasing the staff workforce and economic support systems would lead to a holistic improvement in palliative care.

#### 3.2.2. Social and Resources Support: From Support Family and Patients’ Groups to National Committees

Healthcare professionals and family members involved consistently emphasise the crucial role of addressing social aspects within the care framework. Recognising that palliative care extends beyond the purely medical domain, there is a shared acknowledgement of the impact that social support can have on patients and their families. To address this, there is a strong consensus among families on the necessity of establishing support groups within palliative care units or fostering collaborations with external associations linked to hospitals. These support groups are envisioned as multifaceted resources, serving not only as a source of emotional and psychological solace during the challenging journey of illness but also as invaluable repositories of information. The exchange of experiences and insights within these groups is seen as a means of empowerment, allowing individuals to navigate the complexities of palliative care more effectively. By integrating such social support mechanisms, healthcare professionals and family members envision a more holistic and compassionate palliative care approach that attends not only to the physical aspects of illness but also to the broader dimensions of human experience and connection.

“*…And as a possibility for you to make contact, to give you another resource, as… «There is this association, this other one»… because sometimes it is good that others tell you about their experience or even inform you about where you are going to go… I wish someone had told me about this…*”

(FC 1).

“*…Imagine that through the palliative team, you could have access to being told «look, I am going to put you in contact with this association that also works in your issue», or «I am going to give you the name of the person who is working with relatives who are in a similar situation» and you decide whether to contact them*”

(FC 1).

The burden of palliative care extends beyond the families and patients directly affected, with healthcare professionals shouldering a significant weight in the process. It is imperative to recognise and address the needs of these professionals who play a pivotal role in delivering compassionate and comprehensive care. Healthcare professionals openly expressed the challenges they face, highlighting the stress and anxiety inherent in their work. Managing the emotional aspects of caring for patients with life-threatening illnesses adds a layer of complexity to their responsibilities. The emotional toll of witnessing the struggles and suffering of patients, coupled with the intense demands of the caregiving environment, underscores the importance of supporting healthcare professionals in their crucial roles. Addressing their well-being and providing resources for emotional resilience is not just a matter of professional development but is intrinsic to sustaining a healthcare system that can deliver high-quality, empathetic palliative care. Recognising and mitigating the burden on healthcare professionals contributes not only to their well-being but also ensures the continued delivery of effective and compassionate palliative care for patients and their families.

“*… there is no professional quality of life… In internal medicine, we used a questionnaire that measures the risk biopsychosocial and did not pass eh because for a very specific circumstance and there was measured the level of stress and we came out a stress level of ninety, an exaggerated thing, but usually do not pass any questionnaire…*”

(HP, N1).

”*The level of anxiety is very high and close to depression is not far away.” “We do what we can, we try to take care of ourselves… it’s indeed very complicated, very complicated*”

(HP, N9).

The healthcare team claimed that before the COVID-19 pandemic, they were followed up by a questionnaire on professional satisfaction to keep in mind that the professional should be considered and taken care of. Retaking these questionnaires could be a good proposition.

“*Covid came, and all got jammed… I take it for granted. Of course, you have stress, anxiety, and depression, of course, you do, but, but we don’t measure it, I think it should be measured… and we should have more resources to be able to do everything and do it well, and not doing this is not doing things well*”.

(HM, M8).

Following this idea, some of the medical team also proposed the creation of committees in the national care system to make an adequate follow-up on the satisfaction and state of health of the professionals. Some ideas, above the creation of these units, could be to retake the questionnaires and control support sessions—as it was used before the COVID pandemic—and commit to acquiring necessary resources in palliative care units.

“*…There are things that are already measured and that are known from SECPAL—the Spanish Palliative Care Society—which has already mapped out the necessary resources…it is already known that there should be one home care team for every hundred thousand patients, right now… we are far short of meeting this target, far short because, at the beginning of the creation of the comprehensive plan, of the regional palliative care plan, it was done equitably to the number of health cards and how many support teams were needed. That was fourteen years ago… and the population has logically increased, so now there is a huge disparity between areas. Some areas have grown more and right now the resources are insufficient in all of them…*”

(HM, M3).

The common theme across these discussions is the central role of a comprehensive support system, not only for patients and their families but also for healthcare professionals. Such support should encompass psychological, emotional, socio-familiar, administrative, and economic aspects. The burden of care is, in professionals’ words, the main reason why a patient is hospitalised in a final situation and not before. “*There is an indissoluble union among patient, family and professional caregiver*” (HM, M8).

#### 3.2.3. Upgrading in the Professional Procedures

Improvements in the realm of the palliative care system should also extend to the way healthcare professionals operate within the system. The effectiveness and quality of healthcare and palliative care system are intrinsically linked to the knowledge, skills, and practices of healthcare providers. Among the initiatives to enhance healthcare standards stand the coordination between healthcare institutions and social services. This lack of coordination has left family members feeling abandoned. They highlight the importance of better cooperation between these entities:

“*A bit of abandonment of the system, of the transition…the transition when you are referred to palliative care, which is also a very complicated decision for them, even their family doctor, in a way, when he found out that we wanted this, he gave up a bit, as if he didn’t agree…and it was as if he didn’t want to know anything about the palliative care area, right? so of course…the coordination of the system is for me what failed the most*”

(FC 1).

Another critical aspect of professional procedures is the registration and systematisation of grief in clinical reports. While some professionals currently provide post-death support to families and believe this task is an essential part of palliative unit care work, this information is not systematically registered, leading to an under-documented account of this crucial aspect within the system. The bereavement of grief is a profound experience for families, and acknowledging and recording this process systematically is crucial for understanding the impact of palliative care.

Therefore, early engagement and better coordination with social services, along with the creation of grief care reports, are essential steps to upgrade the palliative care system, positively impacting patients and their families.

### 3.3. A Family Caregiver Experience in Paediatrics Palliative Care System: A Field to Explore

While many improvement suggestions are relevant to palliative care in general, it is crucial to recognise that palliative paediatrics has unique characteristics and specific needs due to the young age of the patients with life-limiting conditions. The insights into improvements in the paediatric palliative care unit were obtained thanks to the participation of the family’s focus groups and the in-depth interview. Focusing on a specific case is not intended for generalisation but rather to provide a rich and illustrative example of a paediatric palliative care system. This approach appreciates the significant information garnered from the individual case while also acknowledging the need for future efforts and advancements in this field.

#### 3.3.1. Same Problems but Different Intensities

Families in paediatric palliative face similar issues to those in adult palliative care but with distinct intensities. Resource scarcity and lack of available healthcare personnel generate difficulties for families who require constant care. The absence of paediatricians on weekends in the region of Murcia adds to the challenges. This situation has led paediatricians to share their personal contact information with families to provide urgent assistance.

“*…Because the problem that Murcia had at that time was that it only had three teams to be able to attend. I think there are fifty or so families in a region where you have families ninety kilometres away like Yecla or any other place. Sometimes they didn’t have the time to get there or to be on the phone to be able to assist you. So, the Murcia team…they gave their telephones…*”

To address this problem, they propose implementing rotating paediatrician teams and ensuring 24 h access to paediatric specialists (FC 4).

“*… They said that the best system was to rotate all of them at the same time so that they could cover the weekends…*”

(FC 4).

“*What does it take to have a 24-h on-call service? That is the essence…a team that is there, on duty, in the afternoons, evenings and weekends you need five teams for that to be effective…but if you only have one…that’s what happens in Murcia…*”

(FC 4).

The coordination issues are also extended to paediatric palliative care, with families reporting greatly suffering from the absence of communication channels between paediatricians, specialists, and other essential units of the healthcare system. For example, they inform that 112 (emergency call number in Spain) does not have the expedient for kids in palliative care, so if an emergency enters the system, families are not attended to correctly, leaving them in dire situations.

“*… If we call [to 112 number] it is because our palliative care team cannot come and because we need them to be attended directly by the doctor or directly taken to the hospital…we are in a very serious situation. A mobile unit or ICU unit must come, a normal ambulance cannot come…*”.

“*Doctors speak directly with the specialists… in an admission, for example, there is direct communication… if there are any doubts or such… palliative or home doctors speak with the specialists to modify the treatment for everything [not to paediatricians]*”

(FC 4).

Another key to paediatric palliatives is emphatic support. Childhood diseases often involve complex medical conditions in a very early moment of life. Children in palliative care not only require excellent medical attention but also need emotional and psychological support, including attention to their families who navigate the challenging journey alongside them. This level of specialised care requires a team whose empathy and experience extend far beyond traditional healthcare.

“*…In summer, substitutes came in, but people who did not have much experience came… we had nurses who came from nursing homes who had not even been in a hospital, and they trembled when they had to do anything*”

(FC 4).

However, support means beyond the professionalism, experience, and human treatment of the professionals. It also means focusing on the special caregiver burden that families in paediatric palliative face. Having a kid in paediatric palliative units means giving up work and consequently reducing their family’s financial income. This overburdening of the caregiver can lead even to the development of diseases by neglecting their health (e.g., not attending a mammography appointment).

“*If I have to go two nights without sleep, three nights without sleep, until [she names her daughter] comes out of that risk zone… of course you get overloaded*”

(FC 4).

“*…I had to give up law… I had three contracted lawyers, a huge office and I have to give it all up, it is impossible to make it compatible. You can’t…”*

(FC 4).

“*…one of the partners does not work, sometimes even both, but normally one is working and the other is in caring. You can’t go to the doctor, you can’t go to buy medicine sometimes, that is if you have… if you have not an extended family or friends around you…*”

(FC 4).

“*…I am self-employed. The issue with so many requirements for a child to enter palliative care… the family has no resources. It is difficult to get direct help. There are fifty families and maybe twenty of those fifty need it… direct help to that family. The problem of getting food to the house or paying for electricity and water must be not a worry*”

(FC 4).

Support for these families must encompass psychological, emotional, socio-familiar, administrative, and economic aspects too. This support needs to be not only provided but also delivered promptly and directly.

“*Because this is not going there, ask for aid and have it… aid that is going to reach you after eight months or when your child has already died*”

(FC 4).

#### 3.3.2. Particular Challenges in Paediatric Palliative Care

In paediatric palliative care, there are unique challenges beyond those faced by adults.

Families expressed the need for continuity in the composition of their care teams due to the close bonds that form between families and their medical teams. Changes in the care team can disrupt the trust and require unnecessary effort to start by becoming familiar with the needs of the family and the patient's situation.

“*…In November of that year, they said that they were removing everyone, and new people were coming in without any training, without having been in palliative care, and without knowing the families and the children here. During this time, you create a bond with the paediatricians and that is very important because not all families know how to communicate in the same way. Not all families for example speak fluent Spanish like us, or the children don’t. They can’t express everything. Each family has a specific need…*”

(FC 4).

Regarding changes in the configuration of the medical team, it is essential to handle these transitions with sensitivity and minimal disruption. The lives of both patients and families are already greatly shaken by the illness challenges they are facing, so dramatic adjustment can exacerbate the difficulties they encounter.

There is also a common demand for addressing the substantial gap that exists between paediatric palliative care units and adult palliative care units; as well as between home care palliative care units and hospital-based units. Families advocate for the establishment of transitional units.

“*My daughter is now eighteen years old. We are still in paediatrics because she is still an oncological child and is a complex child and well, we still have a few days to go to adults. The same thing happens with children who are in palliative care and are old enough to go to adult palliative care. We need a transition team, a team between paediatrics and adults*”

(FC 4).

“*That child goes to home care and the truth is that the home care service has nothing to do with paediatrics’… if there was a transition team… that family with that child… It would make everything much easier for the family as well*”

(FC 4).

Another specific care involving the units’ requirements is about expanding “complex units”. These units are created for children with rare and life-threatening illnesses. These facilities should not be considered exceptions, as their contribution to both the families and patients is profoundly significant. Complex units offer a unique and invaluable solution, providing concentrated attention and specialised treatment within a single care facility, thereby eliminating the need for constant transfers and consultations with various specialists. Is a more individualised treatment that functions, in the eyes of family caregivers, as a “protective bubble” (FC 4).

## 4. Discussion

The predominant research in this field tends to be qualitative studies, primarily due to the challenges associated with accessing representative samples and the complexities involved in studying the needs of patients and families facing life-threatening illnesses. Nevertheless, we aim to complement this prevailing trend by incorporating quantitative studies into this discussion. This approach seeks to strengthen the robustness of the results obtained in our study, challenging the notion that our stakeholders are solely the ones experiencing dissatisfaction and serving as the primary motivation for their participation.

Professionals in the VAUCH team acknowledged the prioritization of physical symptoms over psychological aspects due to time constraints, workforce shortages, and inadequate institutional support, as well as the deficiencies in their training, as reported in another study within VAUCH [27]. Families not only in this research but also in previous studies conducted in VAUCH, especially in the field of neonatal care grieving [14], echoed this sentiment, feeling abandoned within a treatment approach lacking in social and emotional dimensions. This is a clear error due to the implication fact that has been shown: the alleviation of anxiety and resolution of depression, as well as the provision of verbal and nonverbal support, positively influences the quality of dying of palliative patients [28]. Moreover, research also indicates a notable reduction in the burden on family caregivers when these emotional and psychological dimensions are adequately addressed [29].

As a result, families expressed in our research some changes in the support they received as well as some specific demands such as the enhancement of the association role in the healthcare system. Social support is remarked as fundamental in quantitative studies [30,31]. A review of community-based palliative care (CBPC) programmes [32] found that programmes based on social networks and efficient coordination between community, home-based care programmes, and primary health care could improve patients' life quality as well as caregiver burden. This approach could be one of the responses to address some of the deficiencies we detected, such as the scarcity of workforce (of healthcare professionals but also social workers) and inadequate resources for a more comprehensive treatment. Previous studies [31,32] have reported the positive impact of social support in reducing hospitalisations. The provision of social support to families and patients serves as a buffer against stressors, enhances coping mechanisms and provides essential assistance in patient care. This approach results in a notable reduction in caregiver burden, leading to an improvement in overall patient treatment. Importantly, this positive impact extends to a decrease in the frequency of hospitalisations and the duration of hospital stays that could have an impact of over 2% on a country’s GDP [32]. Therefore, when evaluating the feasibility of the recommendations proposed in this research, it is crucial to recognise that financial investment not only aligns with enhancing the quality of palliative care for patients but also contributes to the satisfaction and dignity of professionals’ work conditions while simultaneously health improvements, in turn, result in cost savings for healthcare institutions and the broader healthcare system.

A way to ensure a comprehensive treatment of palliative patients is also providing good quality indicators or scales to evaluate these patients and their needs. In our study, we recollect the distinctive indicators the VAUCH team used and known to evaluate palliative patients, arriving at the final idea that changes should be made in this procedure, too. Specifically, we propose the adoption of the FAMCARE scale with adjustments, emphasising some dimensions, such as the measurement of empathic professional assistance. This modification aligns with findings from studies on the most crucial elements valued by palliative patients and their families [28]. These elements include effective communication and shared decision-making, expert care, respectful and compassionate treatment, and trust and confidence in clinicians. Integrating these considerations, along with insights gleaned from our data (p., e.g., attention to social, psychological, administrative, and economic dimensions) underscores the inadequacy of current scales like Kanorsky, ECOG, Edmonton, or Gijón in addressing the holistic needs of palliative patients. Thus, adopting modified indicators is imperative for accurately capturing and addressing the multifaceted requirements of palliative care recipients. Despite presenting FAMCARE as a promising avenue for enhancing the holistic evaluation of palliative patients, the commitment requires sufficient time from health professionals. Therefore, to ensure the improvement of palliative care for patients and their families, enhancements in work conditions and the availability of resources must be made.

Other aspects of attending are the paediatric palliative needs and the improvements that can be made. As we observed, paediatric palliative patients deal with other intensities of palliative care issues because of the patients' early age. Previous studies on paediatric palliative care units have studied their critical issues and the way to improve them. Among the findings, they remarked the coordination issues between paediatrics and other professionals such as social workers or the rest of the medical team [33,34], the lack of emotional support (during and after de illness [34,35,36], the need of maintaining continuity in the medical team, the constant of attention by paediatrics (24-h attention) due to the special bond and trust created with families [34] and the difficulties related to the transition from paediatric palliative to adult palliative [36], which can suggest the idea of the establishment of transition units.

Finally, there is a need for further research on quantitative studies of the long-term benefits of interventions and a focus on socio-economic aspects. It is also crucial to assess the feasibility of the suggestions and changes in palliative care policies to meet the demands of paediatric palliative care. Above all, some insurgencies should be transferred to hospitals to emphasise the exploration of new applications of the FAMCARE scale and understanding of the associated costs. Further research should attend to these gaps. Additionally, there is a call for incorporating the bereavement and grief aspects into clinical reports.

## 5. Conclusions

This research has highlighted several aspects of VAUCH’s palliative care system, pointing out the shortage of healthcare staff and resources to comprehensively assess and address the needs of palliative patients and their families. It also pointed to the need to establish the use of improved indicators to measure the quality of care and assistance and identified problem areas in palliative care services, as well as proposals for improvement.

Several suggestions have been put forth by healthcare professionals and families affected by a system that has often left them feeling abandoned. These recommendations span from providing holistic care—giving attention to the psychological, emotional, socio-familiar, administrative, and economic aspects of care—to expanding the creation of complex units and establishing new ones or strengthening the connection with palliative care associations. All these recommendations should be thoroughly examined in the context of paediatric palliative care due to their unique challenges and characteristics. Furthermore, it is essential to incorporate some of the families’ petitions, such as maintaining continuity in the medical teams or establishing transitional units. Addressing these areas of improvement is crucial to providing more comprehensive, patient-centred, and empathetic palliative care for both adults and children.

Our research has gone beyond merely identifying the problems within the palliative care system; it has provided valuable insights that have become points for better health policies and the necessity to implement modifications for a more comprehensive evaluation of palliative patients. Furthermore, the findings from this study should be applied to other hospitals to discern common issues and determine the most effective suggestions, considering the varying resources available in each case.

## 6. Strengths and Limitations

The study shows some deficiencies in the palliative care systems and contributes to the policy field with some suggestions and ideas for implementation that can be made in the palliative care system. These suggestions have been conveyed to hospital policymakers and policymakers of the regional healthcare system. This proactive dissemination aims to foster meaningful dialogue and collaborative efforts toward addressing and implementing positive changes in the palliative care landscape.

However, it is important to acknowledge certain biases inherent in the research methodology, particularly related to purposive sampling. While this sampling strategy facilitated access to challenging stakeholders—healthcare professionals grappling with time constraints and families navigating difficult moments—it comes with limitations in terms of representativeness. The sampling process, managed by the clinical team of HCUVA, may have inadvertently led to the inclusion of highly engaged or dissatisfied participants, influencing the generalizability of the findings. Moreover, the specific context of the hospital introduces an additional layer of potential bias. The unique operational and resource aspects of HCUVA may affect the transferability of our findings to various healthcare settings, underscoring the importance of exercising caution when interpreting and applying the study’s outcomes in diverse contexts.

Another notable bias pertains to the homogeneity of the sociodemographic characteristics of the sample, consisting entirely of white Spanish participants. This limited representation raises concerns about the applicability of the findings to individuals from minority ethnic communities. Additionally, the predominantly female composition of the sample (10 out of 12 participants) may not fully capture the experiences of male participants or the unique challenges faced by family caregivers of paediatric patients, as only one such caregiver participated, which also reflects the feminisation of care.

To strengthen the robustness of the study’s findings, future research endeavours should focus on increasing the sample size, ensuring greater sociodemographic heterogeneity among participants, and exploring palliative care units in diverse demographic settings within the country as well as attending more paediatric family caregivers. This approach would enhance the comprehensiveness and generalisability of the research findings, contributing to a more nuanced understanding of palliative care challenges and potential solutions.

## Figures and Tables

**Table 1:** Sociodemographic characteristics of participants (focus groups and interviews).

Healthcare Professionals (HP) n = 9		Family Caregivers (FC) n = 4	
Gender		Gender	
Woman	5 (55.56%)	Woman	3 (75%)
Man	4 (44.46%)	Man	1 (25%)
Average age	47 years old	Average age	54 years old
Professional profile		Professional profile	
Health manager ** (HM)	4 (44.44%)	Education	1 (25%)
Nursing (N)	3 (33.33%)	Law	1 (25%)
Medicine (M)	2 (22.22%)	Housekeeper	1 (25%)
Specialisation		Retiree	1 (25%)
Adults palliative care *	3 (33.33%)	Familiar relationship with the patient	
Paediatric palliative care	3 (33.33%)	Parenthood	3 (75%)
Chronic	1 (11.11%)	Other	1 (25%)
Internist	1 (11.11%)	Palliative unit of their familiar	
Primary care physician	1 (11.11%)	Adult	3 (75%)
Average experience in the area		Paediatrician	1 (25%)
Adults palliative care *	14 years	Pathology of their familiar	
Paediatric palliative care	14 years	Alzheimer	1 (25%)
Chronic	9 years	Multiple morbidities	1 (25%)
Internist	20 years	Oncological	2 (50%)
Primary care physician	22 years		
Training courses in palliative care			
Yes	8 (88.88%)		
No	1 (11.11%)		

* Healthcare participants can work in different units at the same time. ** All of them have a medical profile.

**Table 2:** Main and sub-themes.

Main Thematic Categories	Sub-Thematic Categories
Palliative care indicators	Usage; adequacy; proposals
Improvements in adults’ palliative care system	Timing constraints; health personal increase; support of social workers and social services; establishment of commissions; health professional satisfaction; health professional overburden; institutional coordination; support groups; bereavement support
Improvements in paediatric palliative care system	Health personal increase; empathy; lack of professional training in palliative care; continuity of the care team; caregiver overburden; lack of emotional, spiritual, and psychological support; economic support; 24-h paediatrician care; direct communication among health professionals; units for complex patients, transitional units, and professionals

**Table 3:** Main evaluation indicators of the condition of palliative care patients.

Scale Name	Dimensions	Range	Perceptions of HCUVA Professionals	Use Reason
Kanofsky	Autonomy in the daily task development	0 (autonomous) to 100 (died) [22]	Time-consuming Just palliative patients in an outpatient phase targeted	Brevity
ECOG	Autonomy in the daily task development	0 (autonomous) to 4 (died) [23]	Excessively broad Non-palliative patients targeted	Brevity
Edmonton	physical and psychological alterations related to fragility	0 (non-vulnerability) to 17 (maximum vulnerability) [24]	Extremely long Time-consuming No disease stages covered Lack of significant symptoms (sleeplessness)	Includes interesting symptoms variables: fatigue, nausea, depression, anxiety, loss of appetite, etc.
Gijón	Socio-familiar and economic situation	0 (absence of social problem) to 25 (social problem) [25]	Non-palliative patients targeted	Includes socio-familiar support aspects
FAMCARE	Communication between family/patient and healthcare professional Support: familiar, economic, social, spiritual, and psychological Physical symptoms treatment Availability of care and assistance Family/patient participation in decision-making	20 (unsatisfied) to 100 (totally satisfied) [26]	Palliative patients targeted Lack of empathic professional assistance dimension Suggestions for its incorporation with modifications	Palliative sensitivity

Note: Scale could vary depending on the version of the indicator application.

## Data Availability

Data are contained within the article.

## Supplementary Materials

The following supporting information can be downloaded at: https://www.mdpi.com/article/10.3390/healthcare12010065/s1. Table S1: Consolidated criteria for reporting qualitative studies (COREQ): 32-item checklist.
