# Supplementary material for: Enhancing the Adult and Paediatric Palliative Care System: Spanish Professionals’ and Family Caregivers’ Suggestions for Comprehensive Improvement
Source: Healthcare (Basel). 2023 Dec 27;12(1):65. doi: 10.3390/healthcare12010065 (PMC10779096; doi:10.3390/healthcare12010065)
Supplement: Supplementary file 1 [file healthcare-12-00065-s001.zip › healthcare-2758618-supplementary.pdf]

Table S1. Consolidated criteria for reporting qualitative studies (COREQ): 32-item checklist.

No	Item	Guide questions/description
Domain 1: Research team and reflexivity		
Personal Characteristics		
1.	Interviewer/facilitator	Laura Llop-Medina (LLL)/ Francisco Ródenas-Rigla(FRR)
2.	Credentials	PhD candidate/PhD
3.	Occupation	Researchers at the University of Valencia.
4.	Gender	Female and male.
5.	Experience and training	The researchers were trained in qualitative research techniques and had worked on similar studies in the past.
Relationship with participants		
6.	Relationship established	No relationship was established between the participants and the researcher prior to the start of the study.
7.	Participant knowledge of the interviewer	Participants were provided with an information sheet with the purpose of the study and the researcher's credentials.
8.	Interviewer characteristics	Interviewer name, professional affiliation and education were provided.
Domain 2: study design		
Theoretical framework		
9.	Methodological orientation and Theory	Thematic framework analysis, an inherently comparative form of thematic analysis that employs an organised structure of inductively and deductively derived themes (i.e. a framework) to conduct a cross-sectional analysis through a combination of description and abstraction of data.
Participant selection		
10.	Sampling	Participants were purposively selected meeting thesecriteria:<br>a) Professionals: experienced palliative care manager or professionals b) Family caregivers with current or deseased patients cared for in PCUs

No	Item	Guide questions/description
		Hospital Clínico Universitario Virgen de la Arrixaca. The caregivers chronically ill older patient cared by the home hospitalisation unit of La Fe hospital. The professionals had to be providing direct care to palliative care/chronic patients or managing palliative care services.
11.	Method of approach	Participants were initially identified by members of the hospital's clinical team whitin research team. They arranged the focus group with families by telephone, and by mail with professionals.
12.	Sample size	13 participants: 9 healthcare professionals and 3 family caregivers (focus group.) One more family caregiver participated in a in-depth interview
13.	Non-participation	5 family care givers refused to participate due to the recent death of the patient. 3 professionals refused because of workload.
Setting		
14.	Setting of data collection	All the individual interviews were conducted face to face at University of Murcia.
15.	Presence of non-participants	There was no one else present besides the participants and researchers.
16.	Description of sample	See Results section.
Data collection		
17.	Interview guide	Interview scripts were provided by the authors, and a pilot test was conducted with a patient and a caregiver to ensure that the questions were understandable.
18.	Repeat interviews	No.
19.	Audio/visual recording	Data were collected via voice recorder after receiving explicit consent from participants.
20.	Field notes	Field notes were taken during the interviews to assist the researchers in the interpretation of the data collected.
21.	Duration	Focus groups: average duration 95 minutes. Interviews: average duration 40 minutes.
22.	Data saturation	Data saturation was discussed and agreed between the research authors of the manuscript.

No	Item	Guide questions/description
23.	Transcripts returned	Transcripts were not provided but the results were discussed with the participants.
Domain 3: analysis and findingsz		
Data analysis		
24.	Number of data coders	The data were coded by two of the authors of the manuscript (LLL and PGM)
25.	Description of the coding tree	See table 2
26.	Derivation of themes	Themes were identified as derived from data.
27.	Software	Atlas.Ti software was used to manage the data.
28.	Participant checking	The results were discussed with the participants.
Reporting		
29.	Quotations presented	Quotes from participants were presented to illustrate the main findings. Each quote was identified with the number and profile of each participant. HP (health professionals) and FC (family caregivers). Within the group of proffesionals we distinguised: M (medicine) and N (nursing)
30.	Data and findings consistent	The manuscript presents consistency between the data collected and the results described.
31.	Clarity of major themes	The three main themes were presented in the findings in sections
32.	Clarity of minor themes	The 22 sub thematic themes were presented in the section of results.
